# Evaluating Machine Learning Stability in Predicting Depression and Anxiety Amidst Subjective Response Errors

**DOI:** 10.3390/healthcare12060625

**Published:** 2024-03-10

**Authors:** Wai Lim Ku, Hua Min

**Affiliations:** 1 Systems Biology Center, National Heart, Lung and Blood Institute, NIH, Bethesda, MD 20892, USA; wailim.ku@nih.gov; 2 Department of Health Administration and Policy, College of Public Health, George Mason University, Fairfax, VA 22030, USA

**Keywords:** mental health prediction, machine learning, stability, electronic health records, data perturbation, algorithmic bias, survey data analysis

## Abstract

Major Depressive Disorder (MDD) and Generalized Anxiety Disorder (GAD) pose significant burdens on individuals and society, necessitating accurate prediction methods. Machine learning (ML) algorithms utilizing electronic health records and survey data offer promising tools for forecasting these conditions. However, potential bias and inaccuracies inherent in subjective survey responses can undermine the precision of such predictions. This research investigates the reliability of five prominent ML algorithms—a Convolutional Neural Network (CNN), Random Forest, XGBoost, Logistic Regression, and Naive Bayes—in predicting MDD and GAD. A dataset rich in biomedical, demographic, and self-reported survey information is used to assess the algorithms’ performance under different levels of subjective response inaccuracies. These inaccuracies simulate scenarios with potential memory recall bias and subjective interpretations. While all algorithms demonstrate commendable accuracy with high-quality survey data, their performance diverges significantly when encountering erroneous or biased responses. Notably, the CNN exhibits superior resilience in this context, maintaining performance and even achieving enhanced accuracy, Cohen’s kappa score, and positive precision for both MDD and GAD. This highlights the CNN’s superior ability to handle data unreliability, making it a potentially advantageous choice for predicting mental health conditions based on self-reported data. These findings underscore the critical importance of algorithmic resilience in mental health prediction, particularly when relying on subjective data. They emphasize the need for careful algorithm selection in such contexts, with the CNN emerging as a promising candidate due to its robustness and improved performance under data uncertainties.

## 1. Introduction

MDD and GAD are prevalent psychiatric conditions that significantly impact individuals’ mental health and overall well-being [1]. MDD is characterized by persistent feelings of sadness, diminished interest in activities, altered sleep patterns, appetite disturbances, and, in severe cases, thoughts of self-harm or suicide. In contrast, GAD is characterized by excessive, uncontrollable worry about various life aspects, resulting in heightened anxiety, restlessness, muscle tension, and fatigue. If left untreated, these disorders can severely compromise daily functioning, interpersonal relationships, and overall quality of life [2].

Given the substantial societal and economic burdens posed by MDD and GAD, early detection and intervention are of paramount importance [3]. With technological advancements and the proliferation of data, there is a burgeoning interest in harnessing ML to predict and understand these complex conditions. ML, with its capability to decipher intricate patterns within vast datasets, offers a promising avenue over traditional statistical methods.

While ML offers new avenues for understanding mental health through self-reported data, this approach is not without its difficulties. Survey responses, rich with personal insights, are often marred by errors like recall bias and personal interpretation, introducing variability that could affect the reliability of ML predictions [4,5]. Despite recent advances, there is a notable shortage of comprehensive research examining how well these sophisticated ML algorithms can handle such inconsistencies. Closing this gap is vital—our confidence in ML to accurately predict mental health conditions relies heavily on its performance amidst the natural variability of survey data.

Considering this, the current study aims to critically assess the stability of various ML algorithms, including the increasingly popular CNNs [6], against the backdrop of survey data inaccuracies. By simulating a range of subjective response errors and evaluating algorithm performance, we seek to identify the most reliable methods for predicting MDD and GAD in the context of imperfect data.

CNNs have gained prominence in the field of deep learning due to their powerful feature extraction capabilities, particularly from image data [7]. Here, CNNs are applied to analyze patterns in electronic health records and survey data, hypothesizing their potential to identify subtle, non-linear interactions that may signify the onset of MDD or GAD.

Alongside CNNs, this study examines four established ML algorithms: XGBoost, Random Forest, Logistic Regression, and Naive Bayes. Each algorithm is selected for its proven strengths: XGBoost for its performance and flexibility [8]; Random Forest for its accuracy with complex datasets [9]; Logistic Regression for its interpretability in binary classification tasks [10]; and Naive Bayes for its efficiency in high-dimensional spaces [11].

The selection of these algorithms is driven by their diverse methodologies, providing a comprehensive perspective to the prediction task. By amalgamating the strengths of these algorithms, this study aims for a holistic approach to predicting MDD and GAD, ensuring robust and comprehensive findings. This endeavor bridges the gap between cutting-edge ML techniques and the pressing need for early, precise prediction of psychiatric conditions, heralding timely interventions and enhanced patient outcomes.

While ML models, particularly tree-based methods like Random Forest and XGBoost, have shown promise in mental health predictions [12,13,14,15,16,17,18,19], the reliability of these models when processing subjective survey data is yet to be fully established. Subjective data might be susceptible to biases or errors [20], and the robustness of these models in such contexts remains underexplored. 

This study’s core objective is to evaluate the efficacy and stability of these four ML algorithms—and the newly integrated CNN—in interpreting the extensive University of Nice Sophia Antipolis (UNSA) dataset [21]. Their performance will be assessed under various scenarios of subjective response errors, such as memory recall biases, subjective interpretation, and health-related biases. By evaluating their resilience to such noisy inputs, the study aims to discern the more reliable algorithm for mental health prediction tasks involving subjective data.

The insights gained from this research will illuminate the practicality and robustness of ML models in mental health prediction. Furthermore, findings from this study have the potential to catalyze refinements in early detection and intervention strategies for MDD and GAD, ultimately contributing to better mental health outcomes for individuals and the broader community. 

## 2. Related Review

Recent advancements in ML have revolutionized the field of mental health diagnostics. An increasing body of research has explored the nuanced capabilities of ML in uncovering complex patterns within multifaceted health-related datasets, often revealing insights that traditional statistical methods may overlook. Studies have demonstrated ML’s effectiveness in diagnosing psychiatric conditions by analyzing diverse data sources, such as genetic profiles, neuroimaging, patient-reported histories, and social media data [22,23,24,25,26,27,28,29]. Notably, machine learning algorithms like Support Vector Machines (SVMs) and Gradient Boosting Machines (GBMs) have been extensively investigated for their predictive capabilities in mental health applications, reflecting a growing interest in the integration of technology and psychology [24,25,28,30,31,32].

However, the subjective nature of mental health data, including self-reported symptoms, poses a significant challenge to the predictive accuracy of ML models. The quality of input data is paramount, as inconsistencies in symptom reporting—attributable to memory biases, variable self-assessment thresholds, and other subjective factors—can significantly diminish the reliability of ML outputs [5,33]. Researchers have endeavored to mitigate these biases through novel data preprocessing techniques and algorithmic adjustments [34,35,36,37,38,39], while others have explored the use of ensemble methods to enhance model accuracy and stability, thereby bolstering the robustness of predictions against subjective errors [40,41].

Despite concerted efforts, there remains a discernible gap in the existing literature regarding the resilience of various ML algorithms when faced with data perturbed by subjective response errors. This study aims to bridge this gap by not only critically analyzing the performance of several established ML algorithms but also by investigating the potential of CNNs in addressing the unique challenges posed by subjective mental health data.

This research contributes to the discourse by conducting a systematic evaluation of ML algorithms’ performance, particularly when confronted with the inherent subjectivity of mental health data. The subsequent sections will detail the methodology, implementation, and findings of this study, with a special emphasis on the comparative resilience of the chosen algorithms to subjective response errors in the prediction of MDD and GAD. In doing so, this work aspires to underscore the critical need for robust ML applications in mental health diagnostics and to provide valuable insights into the effective integration of technology in psychological assessments.

## 3. Methods

The research methodology of this study is informed by the understanding that real-world data, especially from self-reports, are often imperfect. Drawing on the precedent set by seminal works in machine learning robustness, this study aims to simulate real-world conditions within our dataset to evaluate the resilience of the chosen algorithms. These foundational studies have demonstrated the necessity of testing against perturbed data to truly assess model performance in practical scenarios, a principle that is central to our approach.

**Figure 1:**
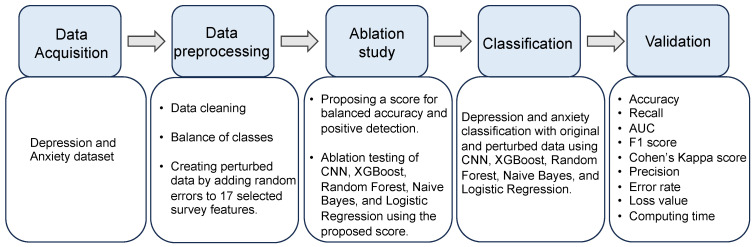
Schematic diagram illustrating the proposed evaluation of machine learning stability in predicting depression and anxiety using five algorithms under varying degrees of subjective response errors.

As illustrated in Figure 1, the research flow of the current study begins with the preparation of the UNSA dataset [21]. To reflect the challenges presented by real-world data collection, we generate perturbed datasets by introducing random errors into 17 selected survey features known to be susceptible to subjective response bias. This perturbation process is quantitatively defined as follows: for each susceptible feature χ with a set of possible responses A , we introduce a random error with a defined probability p , such that a response r is replaced by $U\left(A\right)$ (i.e., a uniformly random selection from the set of all possible answers A for feature χ ), aiming to model the variability and uncertainty inherent in subjective self-reporting. For each feature susceptible to subjective bias, a probabilistic perturbation, defined by the following function, is applied.

$$\left\{\begin{array}{c} \;r,\;with\;probability\;1-p,\\ U\left(A\right),\;\;with\;probabilty\;p. \end{array}\right.$$

This approach is critical to the subsequent analysis, as it mirrors the character-level and word-level input perturbations in natural language processing systems that have been shown to significantly impact model performance [42]. 

The perturbed datasets enable us to perform a comparative evaluation of five distinct ML algorithms—each with varying capacities to process and analyze data—in classifying depression and anxiety. This comparison across unperturbed and perturbed data serves to assess the impact of simulated subjective response errors on the algorithms’ classification abilities, thereby addressing the gap in the literature regarding algorithm robustness in the context of mental health predictions.

In line with the robustness analysis frameworks from related studies [42,43,44], a novel composite score is proposed to identify the configurations that yield the highest accuracy and the most reliable identification of positive cases. This composite score is formulated to weigh the performance accuracy and precision, accommodating the impact of data perturbations, which is paramount in real-world applications where data imperfections are prevalent.

Employing this composite score, we conduct an ablation study to pinpoint the optimal configurations for each algorithm. This step ensures that we compare the algorithms at their highest potential, providing a fair and precise assessment of their respective strengths.

The comprehensive performance evaluation of the algorithms does not stop at accuracy; it extends to a suite of metrics that together provide a multifaceted view of their capabilities. These include recall, precision (both positive and negative), F1 score, Cohen’s kappa score, error rate, loss value, computing time, and the Area Under the Receiver Operating Characteristic (AUC). Each metric offers a unique lens through which to view performance, from general accuracy to the nuances of model reliability and efficiency.

This methodology could provide valuable insights to the field of mental health diagnostics, extending the conversation about the robustness of machine learning algorithms and setting the stage for future research that can build upon our findings to develop even more resilient predictive models.

### 3.1. Participants

The participants in this cross-sectional study consisted of 4184 undergraduate students from the UNSA [21]. Conducted between September 2012 and June 2013, the study included students from diverse faculties, such as sciences, humanities, medicine, law, sports science, engineering, and business. To be part of the study, participants underwent a mandatory medical examination at the university medical service (UMS), except for those summoned for specific reasons like disability management or psychological support. The de-identified dataset, publicly available on Dryad, adhered to strict privacy protocols and was considered observational, obviating the need for written informed consent in accordance with non-interventional clinical research regulations in France. Of the participants, 57.4% identified as female, while 42.6% identified as male, with their ages grouped into four categories: less than 18, 18, 19, and 20 or older, representing 5%, 36%, 28%, and 31%, respectively. The prevalence rates of MDD and GAD were found to be 12% and 8%, respectively. A summary of the participants in this study is shown in Table 1. This table presents an overview of the participant demographics, including sample size, study period, recruitment source, faculty representation, gender distribution, age groups, and prevalence of MDD and GAD.

### 3.2. Measures

The comprehensive measures employed for data collection are fully detailed in the other study [21]. Briefly, the University of Lorraine (France) implemented the CALCIUM software program to standardize data entry and ensure consistent metric collection across a wide array of variables, including demographic information, socioeconomic status, and career-related aspirations [21].

*Standardized Biometric Measurements:* Following established guidelines, trained professionals meticulously measured and recorded biometric variables, including heart rate, blood pressure, BMI, and visual acuity.

*Investigating Mental Health:* Employing a screening process based on clinical experience and the Diagnostic and Statistical Manual of Mental Disorders (DSM-IV) criteria, the study investigated psychiatric symptoms, with a specific focus on depressive disorder, anxiety disorder, and panic attacks.

*Lifestyle Factors and Mental Wellness:* While parental childhood abuse was reported as potentially related to depression and anxiety [45], it was not directly assessed in the questionnaire. Instead, the study focused on individual lifestyle factors, including alcohol consumption, tobacco use, recreational drug usage, and dietary habits, with particular attention paid to unhealthy dietary behaviors, such as irregular meal patterns and unbalanced diets.

*Comprehensive Data for Insight:* The comprehensive data collection aimed to decipher the complex interplay between these diverse factors and the overall well-being of undergraduate university students. This valuable resource promises to offer further insights into their health and mental wellness.

### 3.3. Psychiatric Diagnoses

The investigation into psychiatric diagnoses involved a comprehensive assessment of mental health conditions among undergraduate university students. To determine the prevalence of these conditions, a multi-stage process was implemented. Initially, a screening questionnaire was administered, focusing on four key symptoms of MDD and GAD. For MDD, these symptoms encompassed feelings of sadness or irritability, anhedonia, changes in activity levels, and fatigue or loss of energy. Similarly, the screening for GAD emphasized excessive anxiety and worry, restlessness, fatigue, and irritability. Subsequently, students with positive indications for either MDD or GAD underwent further evaluation to ascertain a complete diagnosis based on the DSM-IV criteria. Qualified medical providers conducted these assessments to ensure accuracy and comprehensiveness. The diagnoses were not viewed in strict binary terms, but rather as dimensional constructs, considering the likelihood of having the disorder based on the presence of at least two associated symptoms. Notably, “anhedonia” and “excessive anxiety and worry” served as core features for depression and anxiety, respectively. Additionally, for the diagnosis of panic attacks, students who reported experiencing at least one panic attack in the past year underwent a rigorous evaluation, requiring the presence of four or more specified symptoms. This thorough examination provided valuable insights into the prevalence and impact of MDD, GAD, and panic attacks among the undergraduate student population, informing the development of targeted interventions and support services tailored to their specific needs.

### 3.4. Data with Unreliability

Survey-based research provides valuable insights into complex mental health phenomena, but ensuring response reliability is crucial when predicting significant outcomes like mental health conditions. This study focuses on 17 specific features within the UNSA dataset known to introduce potential biases in survey responses and which could significantly impact the prediction of mental health conditions (see Table 2 for details). These features cover various areas identified in the literature as susceptible to factors like memory recall errors and health-related biases. By carefully examining these features and their potential biases, the research aims to ensure the data’s reliability and the findings’ accuracy.

*Subjective Interpretation:* Some features are open to personal perceptions and subjective interpretations, which can vary among respondents. These include (1) “difficulty memorizing lessons”, (2) “satisfied with living conditions”, (3) “financial difficulties”, (4) “unbalanced meals”, (5) “eating junk food”, and (6) “irregular rhythm or unbalanced meals”. For instance, perceptions of “satisfying” living conditions can differ widely among individuals. Additionally, familiarity bias, where respondents gravitate towards familiar options, can influence responses, especially in habitual behaviors, as documented by research on product identification [46].

*Memory Recall Biases:* Features that require respondents to recall past behaviors or habits are particularly susceptible to memory recall biases. These features include (7) “long commute”, (8) “irregular rhythm of meals”, (9) “physical activity (3 levels)”, (10) “physical activity (2 levels)”, (11) “cigarette smoker (5 levels)”, (12) “cigarette smoker (3 levels)”, (13) “drinker (3 levels)”, and (14) “drinker (2 levels)”. Schwarz et al. [47] emphasized that respondents often rely on their memory to reproduce previous answers, which can introduce inaccuracies. Borland, Partos, and Cummings [48] further highlighted the susceptibility of certain events to recall biases based on their salience.

*Health-Related Biases:* Features related to health and lifestyle, such as (15) “prehypertension or hypertension”, (16) “binge drinking,” and (17) “marijuana use” can be influenced by social desirability bias. This bias arises when participants present answers that are more socially acceptable than their true behaviors or opinions, especially in health-related contexts [49]. For instance, respondents might underreport substance use due to societal stigmas.

Given the potential biases in these features, data interpretation demands careful consideration. Enhancing survey data reliability and validity necessitates employing strategies like refining instructions, minimizing ambiguities in questions, and cross-validating self-reported data with objective measures. By proactively addressing these biases, this research strives to offer more credible insights into the intricate relationship between lifestyle factors and mental health outcomes.

### 3.5. Data Preprocessing

In this study, missing data were imputed using mean values, and categorical columns were transformed into numerical data using Label Encoding. To create biased datasets, a selected probability of 0.2 was applied to randomly choose students for each of the 17 features considered unreliable. The inputs of the selected students were then converted to exclusive inputs, introducing bias into the dataset. The creation of the biased dataset aimed to explore the impact of unreliable features on predictive models and investigate the robustness of the algorithms in the presence of biased data. 

*Metrics and Composite Score Proposition.* The multifaceted nature of healthcare applications necessitates a comprehensive evaluation of machine learning models’ predictive capabilities. This evaluation employs a suite of metrics, including accuracy, precision, recall, F1 score, Cohen’s kappa, error rate, and AUC.

Accuracy is the most intuitive performance measure, and it is simply the ratio of correctly predicted observations to the total observations. It is useful when the target classes are well balanced. Accuracy is defined with Equation (1).

(1) $$Accuracy=\frac{Number\;of\;correct\;predictions}{Total\;number\;of\;predictions}$$

Recall or sensitivity or the true-positive rate is the ratio of correctly predicted positive observations to all observations in an actual class. It measures the model’s capability to predict the positive cases. Recall is defined with Equation (2).

(2) $$Recall=\frac{True\;Positive}{True\;Positive+False\;Negative}$$

Precision can be seen as a measure of a classifier’s exactness. Positive precision is the ratio of correctly predicted positive observations to the total predicted positives, and negative precision is the ratio of correctly predicted negative observations to the total predicted negatives. Positive precision is defined with Equation (3).

(3) $$Positive\;Precision=\frac{True\;Positive}{True\;Positive+False\;Positive}$$

Negative precision is defined with Equation (4).

(4) $$Negative\;Precision=\frac{True\;Negative}{True\;Negative+False\;Negative}$$

F1 score is the weighted average of precision and recall. Therefore, this score takes both false positives and false negatives into account. It is a better measure than accuracy for imbalanced classes. F1 score is defined with Equation (5).

(5) $$F1\;Score=2\times \frac{Precision\times Recall}{Precision+Recall}$$

Cohen’s kappa ( κ ) is a statistic that measures inter-annotator agreement for categorical items. It is generally thought to be a more robust measure than simple percent agreement calculation, since κ considers the possibility of the agreement occurring by chance. Cohen’s kappa ( κ ) is defined with Equation (6).

(6) $$\kappa =\frac{p_{o}-p_{e}}{1-p_{o}}$$

where $p_{o}$ is the relative observed agreement and $p_{e}$ is the hypothetical probability of chance agreement.

The error rate is the ratio of all incorrect predictions to the total observations and is a complement of accuracy. 

Loss provides a measure of how well the model can achieve the best possible prediction. The term “loss” in this study specifically refers to the function that guides neural network training by measuring the cost of wrong predictions. In this context, it often refers to cross-entropy loss for binary classification tasks. Loss is defined with Equation (7).

(7) $$Loss=-\frac{1}{N}\sum_{i=1}^{N}y_{i}\log\left(p_{i}\right)+\left(1-y_{i}\right)log(1-p_{i})$$

where N is the number of observations, $y_{i}$ is the binary indicator (0 or 1) of the class label for the i th sample, and $p_{i}$ is the predicted probability of the i th sample being of the positive class.

Computing time(s) refers to the computational efficiency of the model, which is critical in real-time applications.

AUC score provides an aggregate measure of performance across all possible classification thresholds. The ROC curve plots the true-positive rate (recall) against the false-positive rate, and AUC represents the area under this curve, quantifying the overall ability of the model to discriminate between the positive and negative classes.

To address the multi-dimensional nature of model evaluation, a composite score ( CS ) is introduced to encapsulate the diverse aspects of performance in a single, coherent framework. The composite score ( CS ) is calculated by integrating the weighted components of the AUC score ( AUC ), the Cohen’s kappa score ( κ ), and the recall ( R ). The formula for the composite score is as follows:

(8) $$CS=\left(w_{1}\times AUC+w_{2}\times \kappa \right)\times H\left(R\right)$$

where $w_{1}$ and $w_{2}$ are the weights representing the importance given to each metric in the composite score, AUC is the area under the ROC curve, κ is the Cohen’s kappa score, R is the recall, and H is the Heaviside step function, where

$$H\left(x\right)=\left\{\begin{array}{c} 1,\;if\;x>0\\ 0,\;if\;x=0 \end{array}\right.$$

The proposed score ensures that a model must excel across all facets of performance, balancing the trade-off between various types of errors, to achieve a high composite score. The exact weights $w_{1}$ and $w_{2}$ can be adjusted based on the relative importance of each metric in the specific context of the application. In this study, the model’s priority lies in high accuracy, with $w_{1}=2\;$ and $w_{2}=0.25$ .

### 3.6. Algorithms’ Description

Consider the input data matrix denoted as $X\in R^{\left(N\times F\right)}$ , where N represents the number of patients and F indicates the number of features per patient. Each element $x_{i,j}$ in the matrix X corresponds to the value of the j -th feature for the i -th patient. 

The anxiety/depression status of the patients is encapsulated as $Y\in {\left\{0,1\right\}}^{N}$ , where each entry $y_{i}$ aligns with a patient and denotes the presence (1) or absence (0) of anxiety or depression.

The aim of this study is to deduce a function G that maps the feature space X to the target variable Y :

$$\hat{Y}=G\left(X\right).$$

Here, $\hat{Y}\in {\left\{0,1\right\}}^{N}$ is the vector of the predicted outcome, striving to closely approximate the actual target variable Y . The effectiveness of the mapping is measured by the proximity of $\hat{Y}$ to Y , often quantified by an appropriate loss function.

Within the framework of neural networks or machine learning models, the function G is ascertained throughout the training phase. Throughout this phase, the model’s parameters are fine-tuned to minimize the loss function, thereby enhancing the model’s predictive accuracy.

Before training the machine learning model, the input data matrix X is divided into two subsets: $X_{train}$ and $X_{valid}$ . This division is made to separate the data used for learning the parameters of the model (training data) from the data used to evaluate the model’s performance (validation data).

Consider $X_{train}\in R^{\left(N_{train}\times F\right)}$ and $X_{valid}\in R^{\left(N_{valid}\times F\right)}$ , where ${\;N}_{train}$ is the number of patients in the training set and $N_{valid}$ is the number of patients in the validation set. Correspondingly, the target variable Y is also divided into $Y_{train}$ and $Y_{valid}$ , matching the division of X . The model then learns a mapping G from $X_{train}$ to $Y_{train}$ , and its performance is evaluated on $X_{valid}$ , comparing the predicted outcomes ${\hat{Y}}_{valid}$ with $Y_{valid}$ .

It is important to emphasize that the parameter configurations and the best composite score, which is denoted as ${CS}^{*},$ are preserved upon the conclusion of the training phase. These configurations are then consistently employed during the prediction phase across all machine learning methods, including but not limited to CNNs. This practice ensures that the integrity of the model’s ability to generalize is maintained from training to application, thus providing a robust measure of the model’s predictive performance in real-world scenarios.

XGBoost. XGBoost is a highly efficient and scalable implementation of gradient boosting. It leverages structured sparsity and parallel computing to boost the performance of decision tree models. Below is a description of the XGBoost algorithm. 

*A. Objective Function (XGBoost):* In the XGBoost framework, the collective set of model parameters optimized during training is represented by θ . This includes the leaf weights across all trees in the ensemble, denoted by w , which are the output scores assigned to each leaf for any given decision tree within the model. The variable θ is therefore a comprehensive representation of the model's learned parameters, encompassing the decision rules at each node and the final predictions at the leaves.

The training process aims to optimize these parameters θ to minimize the overall objective function $\mathrm{Obj}\left(\theta \right)$ . The objective function is a balance between the fidelity of the model to the training data, expressed by the loss function L , and the simplicity of the model, enforced by the regularization term Ω . By tuning θ , XGBoost refines the predictive capacity of the ensemble while preventing overfitting, ensuring that the model remains robust and generalizable.

For the binary classification employing logistic loss, the deviation between the actual and predicted values is captured by the loss function:

$$L\left(y,\hat{y}\right)=-y\log\left(\hat{y}\right)-\left(1-y\right)\log\left(1-\hat{y}\right),$$

where y is the true label (0 or 1) and $\hat{y}$ is the predicted probability of the instance being in class 1.

Consider a tree function $f\left(X\right)$ that consists of M leaves, the prediction for an input X is given by the following:

$$f\left(X\right)=\sum_{m=1}^{M}c_{m}\cdot I\left(x\in R_{m}\right),$$

where $\;c_{m}\;$ is the value predicted by the m -th leaf and I is an indicator function that returns 1 if X falls into the region $R_{m}$ of the m -th leaf and 0 otherwise.

The final prediction ${\hat{y}}_{i}$ is the sum of the predictions of all individual trees of a set of trees ( $f_{k}$ represents the k -th tree) in the model:

$${\hat{y}}_{i}=\sum_{k=1}^{K}f_{k}\left(X_{i}\right),$$

where K is the total number of trees. The objective function of XGBoost combines the loss function L and a regularization term Ω to control model complexity and prevent overfitting:

$$\mathrm{Obj}\left(\theta \right)=\sum_{i=1}^{n}L\left(y_{i},{\hat{y}}_{i}\right)+\sum_{k=1}^{K}\Omega \left(f_{k}\right),$$

where $y_{i}$ is the corresponding true label and ${\hat{y}}_{i}$ is the prediction for the i -th instance. 

Lastly, the regularization term Ω for a tree $f_{k}$ is defined as follows:

$$\Omega \left(f_{k}\right)=\gamma T+\frac{1}{2}\lambda {\left\|w\right\|}^{2}+\alpha {\left\|w\right\|}_{1},$$

where T is the number of terminal nodes in the tree, w is the vector of leaf weights, γ penalizes the number of leaves to control complexity, λ is the L2 regularization term (Ridge Regression), and α is the L1 regularization term, encouraging sparsity in the leaf weights.

*B. Gradient Boosting (XGBoost):* XGBoost refines the gradient boosting method by constructing an ensemble of decision trees sequentially. Unlike random split selection, XGBoost chooses the best split based on the gain that is calculated from the gradient $g_{i}$ and Hessian $h_{i}$ of the loss function. Each tree is built to correct the errors of the preceding ones, and the model is updated at each step to minimize the objective function:

$${\hat{y}}_{i}^{\left(t\right)}={\hat{y}}_{i}^{\left(t-1\right)}+\eta f_{t}\left(x_{i}\right),$$

where η is the learning rate and $f_{t}$ is the new tree added at iteration t .

*C. Model Complexity and Training Process (XGBoost):* Model complexity is regulated by hyperparameters, such as max_depth, gamma, and colsample_bytree. During training, trees are grown sequentially to a specified max_depth and pruned when the improvement in the loss function is not significant (less than gamma).

At each iteration t, the algorithm calculates the gradients $g_{i}$ and Hessians $h_{i}$ for the loss function, which reflect the first- and second-order conditions for optimization, respectively.

The gradient $g_{i}$ is the first derivative of the loss function with respect to the prediction for instance i , indicating the direction of steepest ascent:

$$g_{i}=\partial_{{\hat{y}}_{i}^{\left(t-1\right)}}L\left(y_{i},{\hat{y}}_{i}^{\left(t-1\right)}\right).$$

The Hessian $h_{i}\;$ is the second derivative, providing information about the curvature of the loss function:

$$h_{i}=\partial_{{\hat{y}}_{i}^{\left(t-1\right)}}^{2}L\left(y_{i},{\hat{y}}_{i}^{\left(t-1\right)}\right).$$

These derivatives are crucial for identifying the optimal split points in the trees and for calculating the score assigned to each leaf.

Unlike traditional gradient boosting methods that consider only the reduction in loss, XGBoost evaluates splits using a metric called “gain”, which incorporates second-order information via the Hessians. The gain for a potential split is quantified as follows:

$$\mathrm{Gain}=\frac{1}{2}\left[\frac{{\left(\sum g_{i}\right)}^{2}}{\sum h_{i}+\lambda }\right]-\gamma .$$

Here, λ is the L2 regularization term on leaf weights, which helps to smooth the final learned weights to prevent overfitting. The term γ is a complexity control that penalizes the addition of new leaves to the tree. XGBoost computes the gain for each candidate split and selects the one with the highest gain value. This strategy balances improving the fit of the model to the training data against the complexity of the model.

*D. Ensemble Prediction (XGBoost):* For binary classification, the final prediction aggregates the weighted predictions of all trees, transforming the sum into a probability using the sigmoid function:

$$p=\frac{1}{1+e^{-\sum_{i=1}^{N}\eta f_{i}\left(x\right)}},$$

where N is the number of trees, η is the learning rate, and $f_{i}\left(x\right)$ is the prediction of the i -th tree.

*Random Forest.* Random Forest is a machine learning algorithm that operates by constructing a multitude of decision trees at training time and outputting the class that is the mode of the classes (classification) of the individual trees. It is an ensemble method that is particularly well-suited for complex tasks because it can capture the non-linear relationships between features (represented by input data X ) and the target variable. Below is the mathematical description of Random Forest.

*A. Bootstrap Aggregating (Random Forest):* Given a training set $X_{train}=x_{1},\ldots ,x_{n}$ with responses $Y=y_{1},\ldots ,y_{n}$ , bagging repeatedly ( B times) selects a random sample with replacement of the training set and fits trees to these samples. For b=1,…,B (where B is the number of trees in the forest), a sample ( $X_{b},Y_{b}$ ) is created by randomly selecting n examples with replacement from the original dataset ( X,Y ). A decision tree $f_{b}$ is trained on this bootstrapped sample ( $X_{b},Y_{b}$ ).

*B. Gini impurity (Random Forest):* In predicting binary outcomes as the presence or absence of anxiety or depression, the classes consist of two elements: 1 (indicating presence) and 0 (indicating absence). The Gini impurity for a dataset D is computed as follows:

$$I_{G}\left(D\right)=1-\left(p_{1}^{2}+p_{0}^{2}\right),$$

where $p_{1}$ is the proportion of items labeled with class 1 (presence of anxiety/depression) in the dataset and $p_{0}$ is the proportion of items labeled with class 0 (absence of anxiety/depression) in the dataset.

*C. Decision Trees (Random Forest):* Each tree grows by recursively partitioning the data. At each node of the tree, the algorithm chooses the best split among all features based on the Gini impurity for classification tasks.

For a given node m , representing a region $R_{m}$ with $N_{m}$ observations from X , a set of possible binary splits S of the data is considered. For each s∈S of the node m in the decision tree, we partition the data into $R_{m,left}$ and $R_{m,right}$ , and the Gini impurity of the split $\Delta \left(s\right)$ is computed as follows:

$$\Delta \left(s\right)=\frac{N_{m,left}}{N_{m}}I_{G}\left(R_{m,left}\right)+\frac{N_{m,right}}{N_{m}}I_{G}\left(R_{m,right}\right).$$

Here, $N_{m,left}$ and $N_{m,right}$ are the number of observations in the left and right partitions created by split s and $N_{m}$ is the total number of observations in node m . The goal is to find the split s that minimizes the Gini impurity $\Delta \left(s\right)$ .

The optimal split s* is chosen that minimizes $\Delta \left(s\right):$

$$s*=\arg\underset{s\in S}{\min}\Delta \left(s\right),$$

$$R_{m,left}=R_{m,left}\left(s*\right),R_{m,right}=R_{m,right}\left(s*\right).$$

*D. Feature Selection (Random Forest):* At each split in a tree, a random subset of features is chosen, and the best split is determined using the Gini impurity within this subset of features.

*E. Prediction (Random Forest):* For classification problems, the Random Forest prediction $\hat{Y}$ for an input x is obtained by taking the mode of the predictions of the B trees:

$$\hat{Y}\left(x\right)=\mathrm{mode}\{f_{b}\left(x\right){\}}_{b=1}^{B}.$$

*Naive Bayes:* Naive Bayes classifiers are a collection of classification algorithms based on Bayes’ Theorem. They are particularly known for text classification and spam filtering. The GaussianNB class from sklearn.naive.bayes provides an implementation of the Gaussian Naive Bayes algorithm.

*A. Training (Naive Bayes):* Naive Bayes classifiers are probabilistic classifiers based on applying Bayes’ Theorem with strong (naive) independence assumptions between the features.

Given a dataset with features $X=\left(x_{1},x_{2},\ldots ,x_{n}\right)$ and a binary target variable Y (representing the presence or absence of depression or anxiety), the Naive Bayes classifier first computes the prior probability of each class $P\left(Y\right)$ , which is the frequency of each class in the training set.

For each feature $x_{i}$ and each class y , it calculates the likelihood $P\left(x_{i}|Y=y\right)$ . This is the conditional probability that feature $x_{i}$ appears given that the outcome is class y . The way this probability is calculated depends on the type of Naive Bayes classifier used. For instance, if it is a Gaussian Naive Bayes classifier, the features are assumed to follow a normal distribution.

*B. Prediction (Naive Bayes):* To make a prediction for a new instance $X^{\prime }$ , the Naive Bayes classifier calculates the posterior probability of each class:

$$P\left(Y=y|X^{\prime }\right)=\frac{P\left(X^{\prime }|Y=y\right)\cdot P\left(Y=y\right)}{P\left(X^{\prime }\right)}$$

Since $P\left(X^{\prime }\right)$ is constant for all classes, we only need to focus on maximizing $P\left(X^{\prime }|Y=y\right)\cdot P\left(Y=y\right)$ . Because of the independence assumption, the joint probability can be expressed as the product of individual probabilities:

$$P\left(X^{\prime }|Y=y\right)=\prod_{i=1}^{n}P\left(x_{i}^{\prime }|Y=y\right).$$

Thus, the class with the highest posterior probability is chosen as the prediction.

*Logistic Regression:* Logistic Regression, despite its name, is a linear model used for binary classification tasks rather than regression. It estimates the probability of a binary response based on one or more predictor variables. The Logistic Regression class from sklearn.linear model is utilized for this purpose.

*A. Training (Logistic Regression):* In Logistic Regression, we model the probability that a particular data point belongs to a particular class. The logistic function is used to convert the linear regression output to a probability:

$$P\left(Y=1|X\right)=\frac{1}{1+e^{-\left(\beta_{0}+\beta_{1}x_{1}+\cdots +\beta_{n}x_{n}\right)}}.$$

The coefficients β (including the intercept $\beta_{0}$ ) are learned from the training data by maximizing the likelihood of the observed data, which can be accomplished using algorithms like gradient descent. This process is also known as Logistic Regression training.

The “sklearn” implementation allows for regularization (penalty terms on the size of the coefficients), controlled by the hyperparameters “penalty” and “C”. The “fit_intercept” parameter dictates whether a bias $\beta_{0}$ is included, the “solver” specifies the optimization algorithm, and “class_weight” can be used to handle imbalanced classes.

*B. Prediction (Logistic Regression):* For prediction, the learned coefficients are used with the logistic function to estimate the probability that Y=1 (depression or anxiety present). If the probability is greater than 0.5, the classifier predicts that the instance belongs to class 1 (depression or anxiety present); otherwise, it predicts class 0 (absence).

*Convolutional Neural Network.* CNNs are deep learning algorithms which are particularly powerful for processing data with a grid-like topology, such as images. For the task of binary classification in this study, a 1D CNN was used, capable of capturing temporal features from an input sequence. The CNN was built using the Sequential model from keras.models, incorporating convolutional layers (Conv1D), pooling layers (MaxPooling1D), and dropout layers (Dropout) to prevent overfitting. The network also includes a flattening step (Flatten) before connecting to dense layers (Dense), concluding with a sigmoid activation function to predict binary outcomes. The model is compiled with the Adam optimizer and binary cross-entropy loss function, reflecting the binary nature of the classification task.

The input data $X_{train}$ are passed into the CNN that consists of multiple layers with parameters. The Convolutional Neural Network is obtained in the training process with a total number of epochs T . The optimized set of parameters θ* gives the best composite score CS* .

For each iteration at t<T , $\theta_{t}$ is updated via several steps which are briefly described below, from step A to step E.

- Forward Pass (CNN): Computation of predictions $\hat{Y}$ using current parameters $\theta_{t}$ .
- Loss Calculation (CNN): Computation of the loss $L\left(t\right)$ based on $\hat{Y}$ and true labels Y .
- Backward Pass (CNN): Calculation of gradients $\nabla_{\theta }L\left(t\right)$ .
- Parameter Update (CNN): Adjustment of $\theta_{t}$ using gradients $\nabla_{\theta }L\left(t\right)$ .
- Callback Adjustment (CNN): Update the best model parameter θ* for the next epoch based on the gradients $\nabla_{\theta }L\left(t\right)$ and the composite score $CS_{t}$ at time t and adjust hyperparameters $\phi_{t}$ such as learning rate if a callback condition is met, based on composite score $CS_{t}$ .
- Prediction (CNN): Make predictions after training.

*A. Forward Pass (CNN):* At the t iteration during the training process, ${\hat{Y}}_{train}$ is computed using the input data $X_{train}$ passing through multiple layers. For simplicity, the time-dependence of parameters is ignored within the layers.

*A1. Convolutional Layers (CNN):* For each convolutional layer l with filter size $f^{\left(l\right)}$ , kernel size $k^{\left(l\right)}$ , and input shape I for the first layer:

$$H^{\left(l\right)}=\sigma \left(conv\left(W^{\left(l\right)},A^{\left(l-1\right)}\right)+b^{\left(l\right)}\right),$$

where $H^{\left(l\right)}$ is the output of the l -th convolutional layer, $W^{\left(l\right)}$ and $b^{\left(l\right)}$ are the weights and biases of the layer, σ is the ReLU activation function, and $A^{\left(l-1\right)}$ is the input to the l -th layer (with $A^{\left(0\right)}=I$ for the first layer, where I is the processed input matrix derived from $X_{train}$ ).

*A2. Pooling Layers (CNN):* After some convolutional layers, a pooling operation is applied to reduce the spatial dimensions of the feature maps. In the case of max pooling:

$$P_{i,j}^{\left(l\right)}=\max\left(H_{m,n}^{\left(l\right)}\right),$$

where $P^{\left(l\right)}$ is the output of the pooling operation for layer l , and the max operation is applied over a predefined neighborhood around the position $\left(i,j\right)$ in the feature map $H^{\left(l\right)}$ . The indices m and  n  iterate over the height and width of the pooling window, respectively. The size of the neighborhood (the “pooling window”) and the stride of the operation are hyperparameters.

*A3. Dropout Layers (CNN):* $D^{\left(l\right)}$ represents the output of applying dropout to the l -th layer’s output in a CNN that incorporates dropout layers. Dropout is a regularization technique used to prevent overfitting by randomly setting a fraction of the input units to 0 at each update during training time.

The operation of dropout on a layer’s output can be described as follows:

$$D^{\left(l\right)}=H^{\left(l\right)}⊙M^{\left(l\right)},$$

where $D^{\left(l\right)}$ is the output after applying dropout, $H^{\left(l\right)}$ is the output of the l -th layer before applying dropout, ⊙ denotes element-wise multiplication, and $M^{\left(l\right)}$ is a mask vector left (or matrix, depending on the dimensionality of $H^{\left(l\right)}).$

Each element is drawn from a Bernoulli distribution with probability p (the dropout rate), indicating whether each unit should be kept (1) or dropped (0). The dropout rate p is a hyperparameter that determines the likelihood of an input unit being set to zero. The purpose of $D^{\left(l\right)}$ is to introduce randomness into the training process, which helps to make the model more robust and less likely to rely on any small set of neurons, thereby reducing overfitting. Dropout is only applied during training, not during evaluation or inference, ensuring that the full capacity of the model is used for predictions. 

*A4. Flatten Layer (CNN):* After applying dropout, the Flatten operation converts the tensor into a vector as described earlier. $D^{\left(l\right)}\in R^{\left(N_{train}\times F\right)}$ represents the output from the last Dropout layer. The Flatten operation transforms $D^{\left(l\right)}$ into a vector $A^{\left(fl\right)}$ , where $A^{\left(fl\right)}\in R^{n}$ and $n=N_{train}\cdot \;F$ . 

*A5. First Dense (Fully Connected) Layer (CNN):* The output of the Flatten layer $A^{\left(fl\right)}$ is passed to the first Dense layer, which is computed as follows:

$$Z^{\left(dense1\right)}=W^{\left(dense1\right)}A^{\left(fl\right)}+b^{\left(dense1\right)},$$

where $W^{\left(dense1\right)}\in R^{m\times n}$ and $b^{\left(dense1\right)}\in R^{m}$ are the weights and biases of the layer, respectively; m is the number of dense neurons (“num_dense_neurons”); and n is the size of the input vector $A^{\left(fl\right)}$ . The activation function $A^{\left(dense1\right)}=\mathrm{ReLU}\left(Z^{\left(dense1\right)}\right)$ is then applied, where $\mathrm{ReLU}\left(x\right)=\max\left(0,x\right)$ . 

*A6. Second Dense (Output) Layer (CNN):* The output $A^{\left(dense1\right)}$ from the first Dense layer is input to the second Dense layer, which is computed as follows:

$$Z^{\left(dense2\right)}=W^{\left(dense2\right)}A^{\left(dense1\right)}+b^{\left(dense2\right)},$$

where $W^{\left(dense2\right)}\in R^{1\times m}$ and $W^{\left(dense2\right)}\in R.$ The sigmoid activation function $A^{\left(dense2\right)}=\sigma \left(Z^{\left(dense2\right)}\right)$ is applied to produce the output, where $\sigma \left(x\right)=\frac{1}{1+e^{-x}}$ . ${\hat{Y}}_{train}=1$ if $A^{\left(dense2\right)}>0.5$ , otherwise ${\hat{Y}}_{train}=0$ .

*B. Loss Calculation (CNN):* To evaluate the loss function L, which measures the difference between the predicted output and the true target values, binary cross-entropy is used in the current study as the common loss function.

The binary cross-entropy loss for a prediction ${\hat{Y}}_{train}$ and true label $Y_{train}$ is given by the following:

$$L\left({\hat{Y}}_{train},Y_{train}\right)=-\frac{1}{N}\sum_{i=1}^{N}\left[{\hat{y}}_{train,i}\log\left(y_{train,i}\right)+\left(1-{\hat{y}}_{train,i}\right)\log\left(1-y_{train,i}\right)\right],$$

where N is the number of samples in the dataset.

*C. Backward Pass (CNN):* The gradient of the loss L with respect to a weight W or bias b at layer l by propagating the gradient back through each layer from the output towards the input.

Let us denote the gradient of the loss L with respect to the activation A at layer l and time t as $\frac{\partial L}{\partial A_{t}^{\left(l\right)}}$ . If the activation $A_{t}^{\left(l\right)}$ is a function of the weight $W_{t}^{\left(l\right)}$ and the input to the layer $Z_{t}^{\left(l-1\right)}$ , then the gradient of L with respect to $W_{t}^{\left(l\right)}$ at time t can be computed as follows:

$$g_{t}^{W^{\left(l\right)}}=\frac{\partial L}{\partial W_{t}^{\left(l\right)}}=\frac{\partial L}{\partial A_{t}^{\left(l\right)}}\cdot \frac{\partial A_{t}^{\left(l\right)}}{\partial Z_{t}^{\left(l-1\right)}}\cdot \frac{\partial Z_{t}^{\left(l-1\right)}}{\partial W_{t}^{\left(l\right)}},$$

where $\frac{\partial A_{t}^{\left(l\right)}}{\partial Z_{t}^{\left(l-1\right)}}\;$ represents the derivative of the activation function at layer l with respect to its input and $\frac{\partial Z_{t}^{\left(l-1\right)}}{\partial W_{t}^{\left(l\right)}}$ is the input to the activation function at layer l (which is the output of the previous layer or the input data for the first layer).

Similarly, the gradient with respect to the biases $b_{t}^{\left(l\right)}$ can be computed as follows:

$$g_{t}^{b^{\left(l\right)}}=\frac{\partial L}{\partial b_{t}^{\left(l\right)}}=\frac{\partial L}{\partial A_{t}^{\left(l\right)}}\cdot \frac{\partial A_{t}^{\left(l\right)}}{\partial b_{t}^{\left(l\right)}}.$$

This process is repeated for each layer in the network during backpropagation to compute gradients for all weights, which are then used to update the weights in the direction that minimizes the loss using the Adam optimizer.

*D. Parameter Update (CNN):* The Adam optimizer combines ideas from two other popular optimization algorithms: AdaGrad and RMSProp. It computes adaptive learning rates for each parameter. 

Suppose that α is the step size (also known as the learning rate) and $\beta_{1}$ and $\beta_{2}$ are the exponential decay rates for the moment estimates, typically set to 0.9 and 0.999, respectively. Also, let ϵ represent a small scalar (e.g., $\left(10^{-8}\right)$ ) to prevent division by zero, and let θ denote the parameters of the model, which include the weights $W_{t}^{\left(l\right)}$ and bias $b_{t}^{\left(l\right)}$ at layer l . The term $g_{t}^{\theta }$ is the gradient of the loss with respect to the parameter at timestep t .

Adam maintains two moving averages for each parameter θ , where $m_{t}^{\theta }$ is the first moment (the mean of gradients) and $v_{t}^{\theta }$ is the second moment (the uncentered variance of gradients). The updates are computed as follows:

1. Update biased first moment estimate:
$$m_{t}^{\theta }=\beta_{1}{\cdot m}_{t-1}^{\theta }+\left(1-\beta_{1}\right)\cdot g_{t}^{\theta }.$$
2. Update biased second raw moment estimate:
$$v_{t}^{\theta }=\beta_{2}{\cdot v}_{t-1}^{\theta }+\left(1-\beta_{2}\right)\cdot {\left(g_{t}^{\theta }\right)}^{2}.$$
3. Compute bias-corrected first moment estimate:
$${\hat{m}}_{t}^{\theta }=\frac{m_{t}^{\theta }}{1-\beta_{1}^{t}}.$$
4. Compute bias-corrected second raw moment estimate:
$${\hat{v}}_{t}^{\theta }=\frac{v_{t}^{\theta }}{1-\beta_{2}^{t}}.$$
5. Update the parameters:
$$\theta_{t+1}=\theta_{t}-\frac{\alpha \cdot {\hat{m}}_{t}^{\theta }}{\sqrt{{\hat{v}}_{t}^{\theta }}+\epsilon }.$$

*E. Callback Adjustment (CNN):* The training process with a callback that monitors and saves the best model parameters based on the composite score is as follows:

$CS_{t}=CS\left(\theta_{t}\right)$ is the composite score on the validation set. If $CS_{t}$ is higher than the best composite score observed so far $CS^{*}$ , then update the best score and save the corresponding parameters:

$$if\;CS_{t}>CS*,\;then:\;\;\;CS*=CS_{t}\;,\;\;\theta *=\theta_{t}$$

*F. Prediction (CNN):* During the prediction phase of CNNs, the input data are passed through the network to obtain the output predictions. This phase involves several steps, which exclude the calculation of loss since the goal is not to train the network but to evaluate new data:

1. Input Processing: The input data are preprocessed to match the input size expected by the network and are often normalized or standardized based on the same criteria used during training.
2. Forward Propagation: The preprocessed input is then fed forward through the network’s layers, including convolutional layers, activation functions, pooling layers, and fully connected layers. Since dropout is not used during prediction, all neurons participate in computing the forward pass.
3. Activation Function: The final layer’s activation function is interpreted as the prediction.

### 3.7. Ablation Study

**Figure 2:**
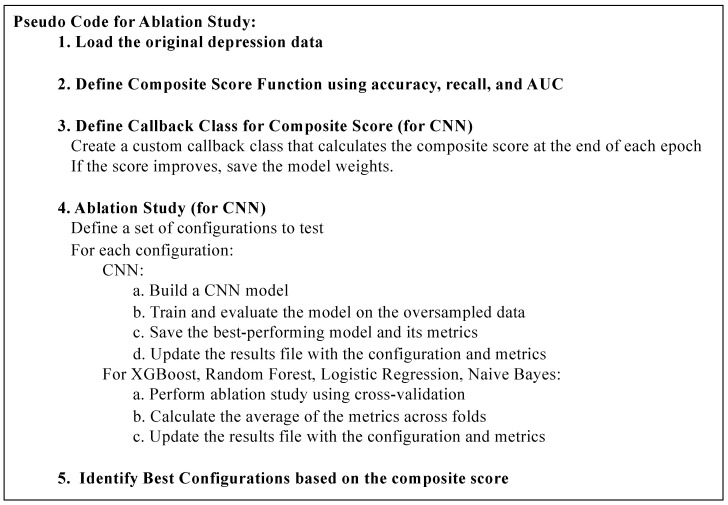
A pseudocode describing the steps of the ablation study. The analysis script is available on GitHub: https://github.com/wailimku/MDD_GAD.git (accessed on 3 March 2024).

This study investigated various parameters across several ML models, including Random Forest, XGBoost, and a CNN. For each parameter, 10-fold cross-validation, a robust method for model evaluation, was utilized to ensure that the findings were not influenced by overfitting or dataset-specific biases. The flow of the ablation study is summarized in the pseudocode shown in Figure 2.

The ablation study culminated in the identification of the most effective configurations for each model. For the CNN model, the optimal settings were: 64 filters, a kernel size of 3, a pool size of 2, dropout utilization with a rate of 0.5, 50 dense neurons, and a single convolutional layer, all processed in batch sizes of 16.

The optimal configurations for the ensemble and linear models were also identified. The Random Forest model performed best with a balanced class weight, 1000 estimators, a maximum depth of 12, a minimum sample split of 12, and a minimum sample leaf of 10. XGBoost achieved superior performance with a binary logistic objective, 50 estimators, a maximum depth of 7, a learning rate of 0.0001, gamma set to 0, alpha at 2.5, and a colsample by tree of 0.2. Logistic Regression yielded the best outcomes with a maximum iteration of 100, L2 regularization, a regularization strength C of 10, fit intercept set to false, a balanced class weight, a solver of Newton-CG, and an L1 ratio of none. Finally, the Naive Bayes model performed optimally with a variance smoothing of 0.0001.

### 3.8. Methods for Imbalanced Data

Several ML methods were employed to address the imbalanced nature of the dataset. The algorithms, including XGBoost, Random Forest, and Logistic Regression, inherently handle imbalanced datasets through their internal mechanisms. For the CNN model, the Synthetic Minority Oversampling Technique (SMOTE) [50] was applied to the original dataset to address the class imbalance before feeding it into the model.

### 3.9. Model Training and Validation

**Figure 3:**
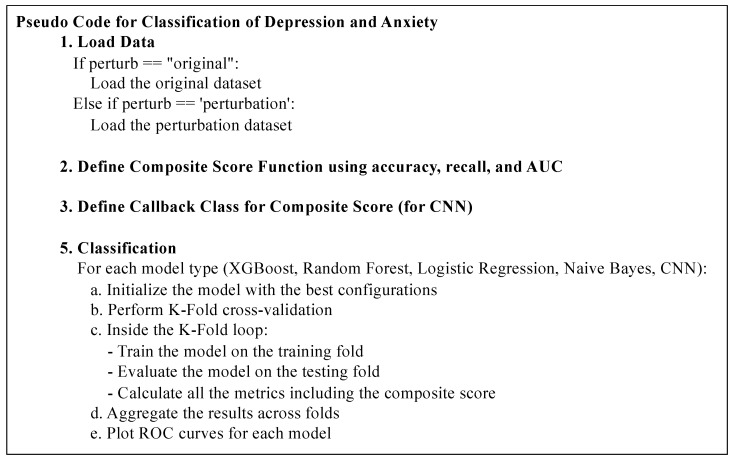
A pseudocode describing the steps of classification of depression and anxiety. Code availability: the analysis script is available on GitHub: http://github.com/wailimku/MDD_GAD.git (accessed on 3 March 2024).

To evaluate the performance and generalizability of the classifier models, a rigorous cross-validation technique was employed. Specifically, a 10-fold cross-validation across the dataset was utilized, as depicted in Figure 3. This method involves partitioning the dataset into 10 equal or nearly equal subsets. In the process of cross-validation, each subset is used once as the validation set while the remaining nine subsets form the training set. Thus, for each validation fold, the data are split in a ratio of 0.1 for validation and 0.9 for training.

This 10-fold cross-validation approach ensures that every data point is used for validation once, allowing us to comprehensively assess the model’s predictive performance. Additionally, this method mitigates the risk of overfitting and provides an unbiased estimate of model performance, as each fold provides an independent test of the model’s ability to generalize to unseen data.

By leveraging the entire dataset for both training and validation, the statistical validity of the results is enhanced. The use of cross-validation is particularly pertinent in studies like the current study where the objective is not only to achieve high predictive accuracy but also to ensure the model’s robustness and reliability in various data scenarios.

### 3.10. Model Evaluation

Additionally, Shapley Additive Explanations (SHAP) scores [51] were utilized for XGBoost and Random Forest to visualize and interpret feature importance within the original data. This comprehensive analysis was conducted to enhance understanding of the models’ predictive performance and robustness under various conditions.

### 3.11. System Configuration for Model Training

To ensure the transparency and reproducibility of our findings, a detailed overview of the system configuration used for training and evaluating the models is described below. The computational environment consisted of a server without a Graphics Processing Unit (GPU), equipped with 20 Central Processing Units (CPUs) to facilitate parallel processing. The system was supported by 1 terabyte (TB) of RAM to efficiently handle large datasets and complex computations without memory constraints. The operating system deployed was Red Hat Enterprise Linux (RHEL) 8/Rocky Linux 8, providing a stable and secure foundation for running our machine learning experiments. All models were implemented and executed using Python version 3.11.0, leveraging state-of-the-art libraries for data manipulation, machine learning, and statistical analysis.

This configuration underscores our commitment to utilizing robust computational resources to ensure the accuracy and reliability of our model evaluations, even in the absence of GPU acceleration.

## 4. Results

*Ablation study of the ML algorithms*. This study investigated the impact of individual parameter modifications on the predictive power of the ML algorithms, focusing on the composite score defined in the Section 3. This score is crucial for evaluating both the models’ ability to distinguish between classes (discriminative ability) and identify positive cases (sensitivity). Due to the greater number of positive cases, the original depression dataset was used for the ablation study.

**Figure 4:**
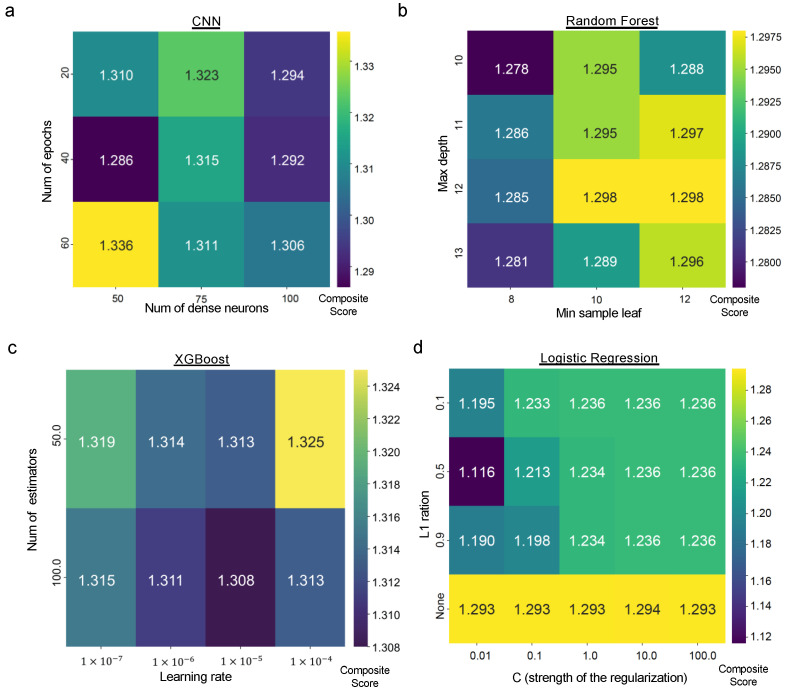
Heatmaps illustrating the impact of hyperparameter tuning on model performance. These heatmaps visualize how strategic adjustments to key hyperparameters affect the model’s performance, measured by the composite score defined in the Section 3. Each heatmap corresponds to a different ML algorithm: (**a**) CNN, (**b**) Random Forest, (**c**) XGBoost, and (**d**) Logistic Regression. The color intensity in each cell represents the corresponding composite score, with brighter colors indicating higher performance. These heatmaps demonstrate that strategic hyperparameter tuning can significantly improve model performance, highlighting the importance of careful parameter optimization for achieving optimal results.

The analysis revealed that specific parameters played a significant role in driving model performance. Figure 4 exemplifies how strategic hyperparameter tuning can significantly improve performance. For CNNs, adjustments to the number of epochs and the size of dense layers were crucial (Figure 4a). The Random Forest model critically relied on modifications to the maximum depth and minimum sample leaf parameters (Figure 4b). In gradient boosting models, variations in the learning rate and the number of estimators demonstrated notable effects on the outcome measures (Figure 4c). Finally, for Logistic Regression, the L1 ratio and C parameter exhibited a significant influence on performance (Figure 4d).

The best configuration for each algorithm, as described in the Section 3, was used for the classification study in both the original and perturbation datasets.

### 4.1. Predictive Performance for the Original Dataset

**Figure 5:**
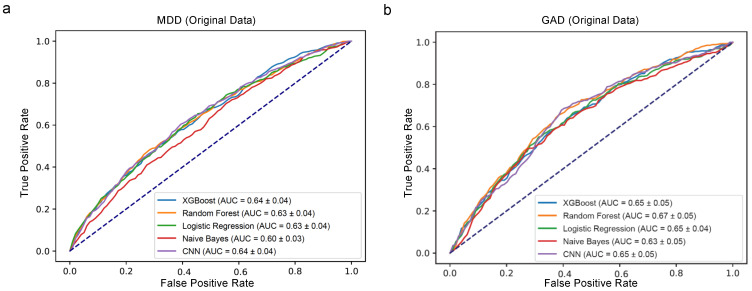
Plots displaying the AUCs for the prediction of (**a**) MDD and (**b**) GAD in the test set, without the introduction of any random errors. The predictions were generated using various models, including Random Forest, XGBoost, Logistic Regression, Naive Bayes, and CNN. The curves show the averaged sensitivity and specificity at different thresholds for prediction using 10-fold cross-validation. The diagonal dashed blue line corresponds to a random classifier.

In evaluating the predictive capabilities of various models for MDD and GAD, a CNN was utilized in conjunction with four ML algorithms: XGBoost, Random Forest, Logistic Regression, and Naive Bayes. These models were tasked with interpreting survey data in their unaltered state, absent of any introduced errors. The AUC scores for MDD were closely matched, with XGBoost, Random Forest, Logistic Regression, Naive Bayes, and CNN yielding scores of 0.64, 0.63, 0.63, 0.60, and 0.64, respectively (as illustrated in Figure 5a). Similar patterns were observed for GAD, with AUC scores reported as 0.65 for Random Forest, 0.67 for XGBoost, and 0.65 for both Logistic Regression and CNN (depicted in Figure 5b).

Comparative analysis demonstrated that Random Forest, XGBoost, and CNN provided a slight edge over their counterparts in terms of accuracy, recall, F1 score, Cohen’s kappa, positive precision, negative precision, error rate, loss, and computing time for both MDD and GAD, as detailed in Table 3 and Table 4. Notably, Random Forest yielded the highest positive precision scores (0.3 for MDD in Table 3 and 0.17 for GAD in Table 4), indicative of its robustness in correctly identifying true-positive cases, while the positive precision was slightly lower for both XGboost (0.2 for MDD in Table 3 and 0.13 for GAD in Table 4) and CNN (0.27 for MDD in Table 3 and 0.16 for GAD in Table 4).

While the AUC values across the models were similar, Random Forest, XGBoost, and CNN exhibited superior performance in other metrics. This discrepancy suggests that although AUC is a crucial indicator of a model’s ability to discriminate between classes, it does not necessarily reflect a model’s precision or its balance of false positives and negatives. Therefore, a model may present a high AUC but still underperform in precision or recall, underscoring the necessity of a multi-metric evaluation approach. Consequently, the Random Forest model emerges as a strong candidate for mental health predictive analyses, particularly where the emphasis is placed on precision over sensitivity. Specifically, the effectiveness of Random Forest in mental health prediction was demonstrated in the study by Tate, A.E. et al. [22], where it outperformed other machine learning techniques, including XGBoost, Logistic Regression, Support Vector Machines, and Neural Networks, in predicting mental health problems in adolescence. In alignment with our findings, Ram Kumar, R.P. et al. [23] also reported superior performance of Random Forest over other classifiers, including a CNN, in the context of predicting heart diseases. Although the studies by Tate, A.E. et al. [22] and Ram Kumar, R.P. et al. [23] are situated in different domains—adolescent mental health and heart disease prediction, respectively, the consistent performance of Random Forest across these varied contexts underscores its potential as a versatile tool for medical diagnostics, including our focus on MDD and GAD. Nevertheless, the application of Random Forest should be tailored to the specific characteristics of the dataset at hand, and further research may be necessary to fully understand its suitability across various settings of mental health diagnostics.

**Figure 6:**
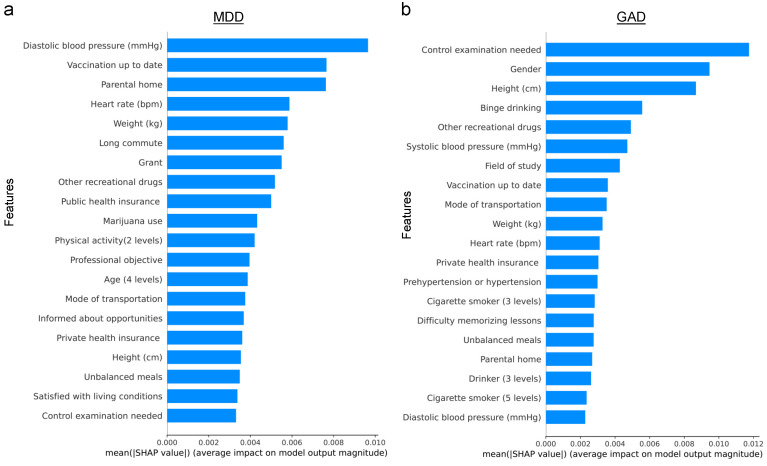
Plots illustrating the importance of features for predicting (**a**) MDD and (**b**) GAD. The plots display the means of the absolute values of SHAP scores across all top-ranked features. A higher SHAP value indicates that the corresponding feature significantly influenced the prediction of the Random Forest model. These plots offer valuable insights into the relative significance of different features in informing the predictions of MDD and GAD, helping to identify the key factors that contribute to the model’s performance.

Next, the assessment of feature importance for predicting MDD and GAD was conducted using the Random Forest model (see Figure 6). Notably, there were no common features among the top five ranked for both conditions. For MDD, the top three ranked features were diastolic blood pressure, up-to-date vaccination status, and parental home. In contrast, the need for a control examination, gender, and height emerged as the top three for GAD. These distinctions are consistent with findings from previous studies, underscoring the unique characteristics associated with each disorder.

**Figure 7:**
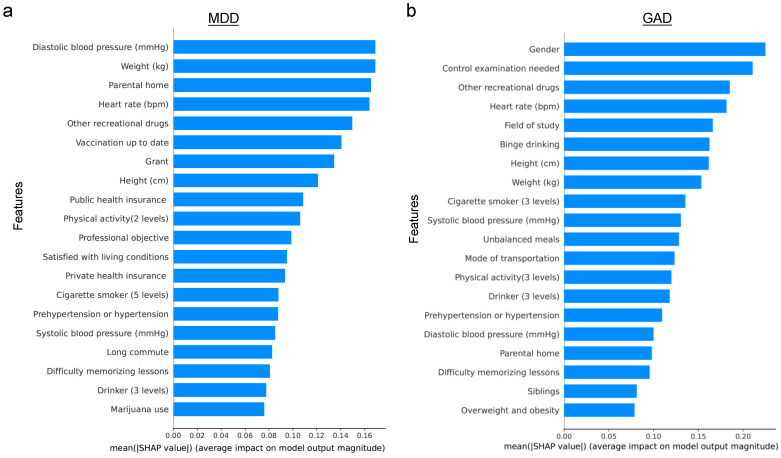
Plots illustrating the importance of features for predicting (**a**) MDD and (**b**) GAD. The plots display the mean of the absolute values of SHAP scores across all top ranked features. A higher SHAP value indicates that the corresponding feature significantly influenced the prediction of the XGBoost model.

Similarly, when evaluating feature importance for predicting MDD and GAD using XGBoost (see Figure 7), a contrast was observed compared to the Random Forest results. Specifically, features such as other recreational drugs and heart rate were shared among the top five ranked features for both MDD and GAD. For MDD, diastolic blood pressure, up-to-date vaccination status, and parental home were of relatively higher importance. In contrast, the need for a control examination, gender, and field of study were more pivotal for GAD prediction. This variation in feature importance between MDD and GAD aligns with the findings from the Random Forest model.

### 4.2. Predictive Performance for the Biased Dataset

Before diving into the robustness test, it is important to acknowledge that some features might have a higher predictive power, necessitating careful consideration. Random Forest identified 5 and 7 features among the top 20 important features that could potentially be prone to bias for MDD and GAD, respectively. On the other hand, XGboost identified 8 and 7 features among the top 20 important features that could be susceptible to bias for MDD and GAD, respectively (see Figure 6 and Figure 7). Recognizing the potential for bias in 17 self-selected features, a robustness test was conducted by introducing random errors into these features, as described in the Section 3.

**Figure 8:**
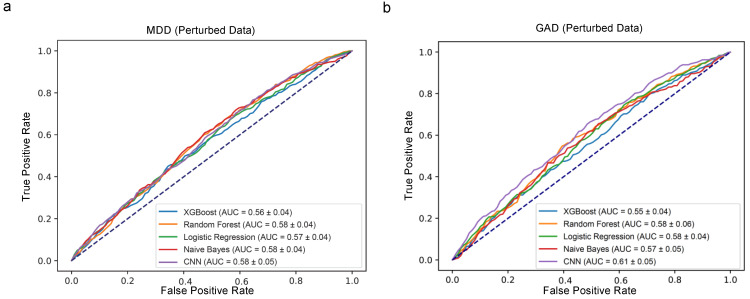
Plots displaying AUCs for the prediction of (**a**) MDD and (**b**) GAD in the test set, with random error introduced to the data of the 17 survey features. The predictions were generated using various models, including Random Forest, XGBoost, Logistic Regression, Naive Bayes, and CNN. The curves show the averaged sensitivity and specificity at different thresholds for prediction using 10-fold cross-validation. The diagonal dashed blue line corresponds to random classifier.

Subsequently, a CNN along with four other ML models—Random Forest, XGBoost, Logistic Regression, and Naive Bayes—were applied to the biased dataset. The resulting AUC scores for MDD exhibited a decline across the board, with scores of 0.56 for Random Forest, 0.58 for XGBoost, 0.57 for Logistic Regression, 0.58 for Naive Bayes, and 0.58 for CNN (as shown in Figure 8a). For GAD, similar reductions were noted, with the AUC values recorded at 0.55, 0.58, 0.58, 0.57, and 0.61 for Random Forest, XGBoost, Logistic Regression, Naive Bayes, and CNN, respectively (illustrated in Figure 8b). These figures represent a marked decrease from the AUC values computed using the original dataset (displayed in Figure 5).

It is important to highlight that, beyond AUC scores, comprehensive metrics revealed distinct model behaviors. Notably, the CNN not only withstood the introduction of random errors but also demonstrated enhanced performance in several key metrics, including accuracy, Cohen’s kappa score, and positive precision, for both MDD and GAD (as seen in Table 5 and Table 6). After the error introduction, the CNN’s positive precision for MDD improved from 0.27 to 0.28, and that for GAD from 0.16 to 0.3. This improvement underlines the CNN’s capability to handle data unreliability, bolstering its predictive precision. In contrast, the Random Forest model’s positive precision plummeted to zero for both disorders, indicating a significant vulnerability to data errors. XGBoost showed a slight decline in positive precision from 0.2 to 0.17 for MDD and from 0.13 to 0.1 for GAD. These outcomes underscore the CNN’s potential advantage in managing survey data fraught with uncertainties.

It is important to understand the impact of subjective response errors on machine learning models used to predict mental health conditions. In this study, emphasis was placed on the CNN algorithm with potential application to other models. A feature-by-feature analysis was conducted, comparing the performance of the CNN on the original dataset and a perturbed counterpart, where each feature was independently infused with a 0.2 probability error. This granular approach allowed us to calculate differences across various metrics, revealing the influence of feature bias on the predictive accuracy for MDD and GAD.

**Figure 9:**
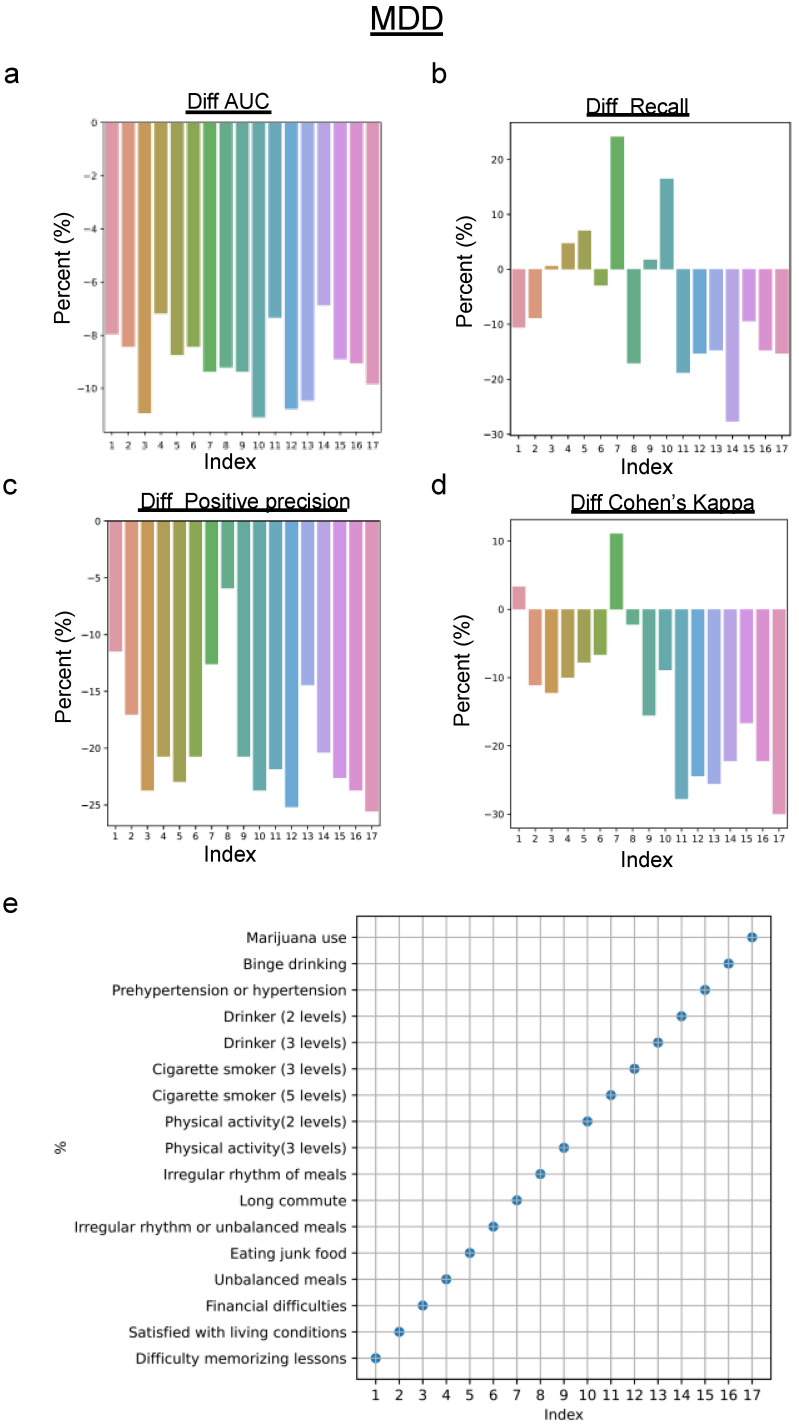
Impact of feature perturbations on MDD prediction performance. This figure shows how perturbing individual features affects CNN’s ability to predict MDD. Each subplot presents a bar chart that compares the change in a specific metric: (**a**) AUC measures the model’s ability to distinguish between true and false positives. (**b**) Recall: the proportion of true positives correctly identified by the model. (**c**) Positive precision: the proportion of predicted positives that are true positives. (**d**) Cohen’s kappa score: a measure of agreement between the model’s predictions and the true labels, considering chance agreement. A negative value in the bar charts indicates a decrease in the model’s performance for that feature when a 0.2 probability error is added. This means that perturbing that feature has a detrimental impact on the model’s ability to accurately predict MDD. (**e**) The scatter plot on the right shows the relationship between the features and their corresponding indices. This helps to identify which features are most sensitive to perturbations and therefore most important for MDD prediction.

**Figure 10:**
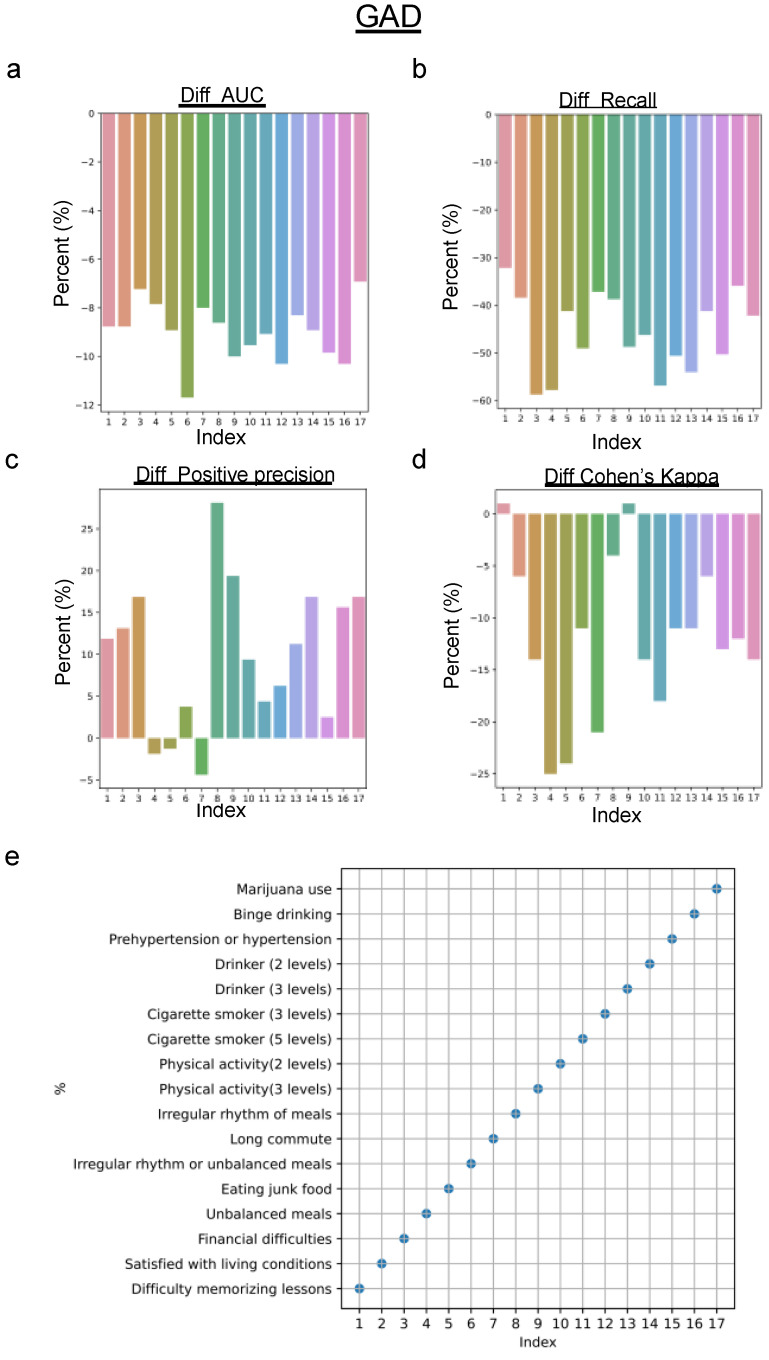
Impact of feature perturbations on GAD prediction performance. This figure shows how perturbing individual features affects CNN’s ability to predict GAD. Each subplot presents a bar chart that compares the change in a specific metric: (**a**) AUC measures the model’s ability to distinguish between true and false positives. (**b**) Recall: the proportion of true positives correctly identified by the model. (**c**) Positive precision: the proportion of predicted positives that are true positives. (**d**) Cohen’s kappa score: a measure of agreement between the model’s predictions and the true labels, considering chance agreement. A negative value in the bar charts indicates a decrease in the model’s performance for that feature when a 0.2 probability error is added. This means that perturbing that feature has a detrimental impact on the model’s ability to accurately predict GAD. (**e**) The scatter plot on the right shows the relationship between the features and their corresponding indices. This helps to identify which features are most sensitive to perturbations and therefore most important for GAD prediction.

For MDD prediction (Figure 9), specific features significantly reduced both AUC score and positive precision, compromising the CNN’s efficacy. One explanation for the discrepancy between the decrease in positive precision with individual feature perturbations and its increase when perturbing all 17 features is that combined errors across multiple features might reduce the misidentification of depression, leading to a paradoxical increase in positive precision. For recall and Cohen’s kappa score, the decreases are mainly related to features with memory recall bias (e.g., drinker and smoker) and health-related bias (e.g., hypertension and bridge drinking), except one feature from subjective interpretation (i.e., eating junk food). Interestingly, the same biases have a less pronounced effect on anxiety prediction (Figure 10), suggesting a differentiated sensitivity within the model’s feature set.

For GAD prediction, different from MDD prediction, perturbation in most single features led to a decrease in recall and an increase in positive precision (Figure 10). Also, different from MDD prediction, a decrease in Cohen’s kappa score was more related to bias in subjective interpretation, especially the feature named “irregular rhythm or unbalanced meals”. This finding highlights the importance of addressing subjective biases in some specific features to ensure the reliability of ML models in mental health assessments.

## 5. Discussion

*Machine Learning’s Rising Influence in Mental Health Prediction:* In recent years, ML methods have gained significant popularity in the field of mental health prediction [22,24,25,26,27]. However, very few studies have evaluated the performance of these ML methods to ensure their reliability and clinical applicability. In this study, a comprehensive analysis was conducted to predict MDD and GAD among undergraduate university students using ML models. The dataset consisted of self-reported survey data from students, covering various socio-demographic characteristics, health-related information, and lifestyle behaviors. Through comparisons of model performance on original and simulated biased self-reported survey data, this study evaluated their reliability.

*Exploring Classifier Performance on Original Survey Data:* The current study evaluated a variety of classifiers, including Random Forest, XGBoost, Logistic Regression, and Naive Bayes, and placed a specific emphasis on CNNs due to their established robustness to data inconsistencies. When applied to the original self-reported survey data, we found that XGBoost, CNN, and Random Forest yielded the highest AUC scores for predicting both MDD and GAD. Notably, Random Forest not only achieved high AUC scores but also exhibited the highest positive precision, signaling its acute ability to correctly identify individuals with these conditions. This result is in line with findings from Smith et al. [22], who highlighted Random Forest’s effectiveness in mental health prediction among adolescents. However, this contrasts with reports from Jones et al. [24] and Lee and Kim [25], where gradient boosting and LightGBM outperformed other models, including Random Forest, in similar tasks.

The observed discrepancies may be due to several factors, including variations in data sources, which might contain different feature sets and distributions; differences in model configurations, such as parameter tuning and feature selection; and the specific contexts of the studies, including the demographic characteristics of the study populations. Such factors underscore the importance of considering context when interpreting model performance.

In our analysis, while the leading algorithms demonstrated similar levels of performance, the decision on which model to deploy in practice should consider the characteristics of the dataset at hand as well as the specific operational requirements of the task. For a detailed comparison of model performance in our study, refer to Table 7.

*Evaluating Model Robustness: A Crucial Innovation:* The true novelty of this study lies in its rigorous assessment of model sturdiness against biased data. While all models demonstrated competence on unbiased data, the introduction of random errors resulted in an anticipated decline in performance across all models; however, the CNN’s resilience remained undeterred. It not only withstood the perturbations but also demonstrated a commendable increase in positive precision for both MDD and GAD. This unwavering performance positions the CNN as an exceptional candidate for mental health prediction amidst data uncertainties, supporting its consideration in clinical and research settings.

*Exploring the Role of Physiological and Environmental Factors in Mental Health:* The analysis of feature importance revealed that physiological markers such as weight, height, heart rate, and blood pressure serve as significant predictors for mental health conditions, corroborating existing research [26]. Furthermore, environmental factors, exemplified by the influence of the parental home, emerged as relevant predictors [52,53], underscoring the multifaceted nature of mental health that encompasses both biological and socio-environmental dimensions.

*Toward a Symbiotic Approach: Combining Data-driven Techniques with Human Expression Interpretation:* This research presents parallels with a prior study utilizing facial expressions to predict Depression, Anxiety, and Stress Scale (DASS) levels [54]. Despite methodological and focal differences, both studies share the goal of enhancing mental health diagnostics. Juxtaposing this study’s emphasis on data reliability with the other study’s focus on continuous DASS scores and real-time monitoring suggests the potential for an interdisciplinary approach. This approach could lead to a hybrid predictive model that combines data-driven ML predictions with the nuanced interpretation of human expressions, potentially revolutionizing mental health diagnostics.

## 6. Conclusions

This study underscores the significant potential of ML algorithms for mental health prediction, emphasizing that their effectiveness critically hinges on resilience to data imperfections. The standout performance of CNNs, even amid subjective response errors, underlines the necessity of choosing algorithms wisely for mental health diagnostics. As we advance, the development of ML models must prioritize not only precision but also robustness to the variances inherent in real-world data—a challenging yet essential pursuit for enhancing model refinement and understanding of mental health disorders and ensuring the reliability and applicability of findings. This endeavor promises to leverage ML’s capabilities to offer real benefits for individuals facing mental health challenges.

The methodologies and insights from this study have broader implications for healthcare, suggesting several avenues for future research and application:

- *Disease Prevention:* Utilizing ML to analyze patterns in lifestyle and genetic data could lead to early identification of risk factors for chronic diseases, such as diabetes and heart disease, enabling preventative measures to be implemented sooner.
- *Symptom Prediction:* ML can be applied to predict the onset of symptoms for diseases like Alzheimer’s and Parkinson’s based on subtle changes in behavior or biomarkers, facilitating early intervention.
- *Personalized Treatment Plans:* By analyzing patient data, ML algorithms can help tailor treatment plans to individual needs, improving outcomes in conditions ranging from cancer to depression.
- *Infection Outbreak Prediction:* ML can be instrumental in predicting the outbreak of infectious diseases by analyzing travel, climate, and health data, allowing for timely public health responses.

Additionally, the integration of multimodal data sources promises to enhance the accuracy and robustness of ML algorithms in these applications. There is a compelling need for innovative methodologies to improve the handling and interpretation of subjective data, not just in mental health but across healthcare. Investigating the deployment of these algorithms in clinical settings will be key to understanding their practical utility, including their interpretability by healthcare professionals.

Expanding our focus to include a broader range of health conditions, alongside a rigorous examination of the ethical and privacy aspects of using electronic health records and self-reported data for ML predictions, will be critical. These steps will ensure that ML technologies can be effectively and ethically integrated into healthcare, providing a foundation for innovative solutions to some of the most pressing health challenges.

## Figures and Tables

**Table 1:** Demographic characteristics of undergraduate students participating in the cross-sectional study.

Feature	Description	Value
Sample Size	Total number of participants	4.18 × 10^3^
Study Period	Dates of data collection	September 2012–June 2013
Recruitment Source	University medical service (UMS)	N/A
Faculty Representation	Diversity of academic disciplines	Sciences, humanities, medicine, law, sports science, engineering, business
Gender Distribution	Percentage of female and male participants	Female: 57.4%, Male: 42.6%
Age Groups	Distribution of participants by age	Less than 18: 5%, 18: 36%, 19: 28%, 20 or older: 31%
MDD Prevalence	Percentage of participants diagnosed with depression	1.20 × 10^−1^
GAD Prevalence	Percentage of participants diagnosed with anxiety	8.00 × 10^−2^

**Table 2:** A list of the 17 selected survey features.

Index	Survey Features
1	Difficulty memorizing lessons
2	Satisfied with living conditions
3	Financial difficulties
4	Unbalanced meals
5	Eating junk food
6	Irregular rhythm or unbalanced meals
7	Long commute
8	Irregular rhythm of meals
9	Physical activity (3 levels)
10	Physical activity (2 levels)
14	Cigarette smoker (5 levels)
12	Cigarette smoker (3 levels)
13	Drinker (3 levels)
14	Drinker (2 levels)
15	Prehypertension or hypertension
16	Binge drinking
17	Marijuana use

**Table 3:** Evaluations of five classification models in predicting MDD. The table presents the performance metrics of these models on the original dataset without any added random errors. The metrics include accuracy, F1 score, Cohen’s kappa score, positive precision, negative precision, error rate, loss, and computing time (seconds). The values and errors are the averages and standard deviations calculated over the results of 10-fold cross-validation.

MDD (Original Data)									
Model	Accuracy	Recall	F1 Weighted	Cohen’s Kappa	Positive Precision	Negative Precision	Error Rate	Loss	Computing Time (s)
XGBoost	0.71 ± 0.03	0.43 ± 0.05	0.75 ± 0.02	0.12 ± 0.04	0.2 ± 0.03	0.9 ± 0.02	0.29 ± 0.03	0.69 ± 0.0	0.14 ± 0.02
Random Forest	0.86 ± 0.02	0.11 ± 0.04	0.82 ± 0.02	0.1 ± 0.05	0.3 ± 0.11	0.88 ± 0.01	0.14 ± 0.02	0.52 ± 0.01	2.99 ± 0.11
Logistic Regression	0.62 ± 0.02	0.55 ± 0.08	0.68 ± 0.02	0.1 ± 0.05	0.18 ± 0.04	0.91 ± 0.02	0.38 ± 0.02	0.65 ± 0.02	0.24 ± 0.03
Naive Bayes	0.53 ± 0.05	0.6 ± 0.1	0.6 ± 0.04	0.05 ± 0.02	0.15 ± 0.02	0.9 ± 0.02	0.47 ± 0.05	0.92 ± 0.08	0.04 ± 0.0
CNN	0.82 ± 0.07	0.17 ± 0.13	0.8 ± 0.04	0.09 ± 0.06	0.27 ± 0.1	0.88 ± 0.02	0.18 ± 0.07	0.44 ± 0.08	228.93 ± 4.67

**Table 4:** Evaluations of five classification models in predicting GAD. The table presents the performance metrics of these models on the original dataset without any added random errors. The metrics include accuracy, F1 score, Cohen’s kappa score, positive precision, negative precision, error rate, loss, and computing time (seconds). The values and errors are the averages and standard deviations calculated over the results of 10-fold cross-validation.

GAD (Original Data)									
Model	Accuracy	Recall	F1 Weighted	Cohen’s Kappa	Positive Precision	Negative Precision	Error Rate	Loss	Computing Time (s)
XGBoost	0.75 ± 0.03	0.4 ± 0.07	0.8 ± 0.02	0.1 ± 0.05	0.13 ± 0.03	0.94 ± 0.01	0.25 ± 0.03	0.69 ± 0.0	0.14 ± 0.02
Random Forest	0.9 ± 0.02	0.08 ± 0.03	0.88 ± 0.02	0.06 ± 0.05	0.17 ± 0.07	0.93 ± 0.01	0.1 ± 0.02	0.42 ± 0.01	2.99 ± 0.1
Logistic Regression	0.64 ± 0.03	0.58 ± 0.09	0.73 ± 0.02	0.08 ± 0.03	0.12 ± 0.02	0.95 ± 0.01	0.36 ± 0.03	0.64 ± 0.02	0.18 ± 0.04
Naive Bayes	0.68 ± 0.04	0.5 ± 0.13	0.76 ± 0.03	0.08 ± 0.04	0.12 ± 0.03	0.94 ± 0.01	0.32 ± 0.04	0.74 ± 0.1	0.04 ± 0.0
CNN	0.8 ± 0.1	0.32 ± 0.19	0.83 ± 0.06	0.1 ± 0.03	0.16 ± 0.05	0.94 ± 0.01	0.2 ± 0.1	0.46 ± 0.12	236.47 ± 5.02

**Table 5:** Evaluations of five classification models in predicting MDD. The table presents the performance metrics of these models on the original dataset with random error introduced to the data of the 17 survey features. The metrics include accuracy, F1 score, Cohen’s kappa score, positive precision, negative precision, error rate, loss, and computing time (seconds). The values and errors are the averages and standard deviations calculated over the results of 10-fold cross-validation.

MDD (Perturbed Data)									
Model	Accuracy	Recall	F1 Weighted	Cohen’s Kappa	Positive Precision	Negative Precision	Error Rate	Loss	Computing Time (s)
XGBoost	0.81 ± 0.02	0.13 ± 0.03	0.8 ± 0.02	0.04 ± 0.04	0.17 ± 0.06	0.88 ± 0.01	0.19 ± 0.02	0.69 ± 0.0	0.14 ± 0.03
Random Forest	0.87 ± 0.02	0.0 ± 0.0	0.82 ± 0.02	0.0 ± 0.0	0.0 ± 0.0	0.87 ± 0.02	0.13 ± 0.02	0.49 ± 0.01	2.95 ± 0.02
Logistic Regression	0.6 ± 0.02	0.48 ± 0.08	0.66 ± 0.02	0.05 ± 0.03	0.15 ± 0.03	0.89 ± 0.01	0.4 ± 0.02	0.67 ± 0.02	0.28 ± 0.03
Naive Bayes	0.45 ± 0.05	0.7 ± 0.07	0.52 ± 0.06	0.04 ± 0.02	0.15 ± 0.02	0.91 ± 0.02	0.55 ± 0.05	0.82 ± 0.07	0.04 ± 0.0
CNN	0.84 ± 0.03	0.1 ± 0.08	0.81 ± 0.02	0.06 ± 0.16	0.28 ± 0.16	0.88 ± 0.02	0.16 ± 0.03	0.44 ± 0.04	252.11 ± 5.38

Note: CNN shows better performance in Positive Precision (0.28 ± 0.16) and Error Rate (0.16 ± 0.03) for MDD perturbed data.

**Table 6:** Evaluations of five classification models in predicting GAD. The table presents the performance metrics of these models on the original dataset with random error introduced to the data of the 17 survey features. The metrics include accuracy, F1 score, Cohen’s kappa score, positive precision, negative precision, error rate, loss, and computing time (seconds). The values and errors are the averages and standard deviations calculated over the results of 10-fold cross-validation.

GAD (Perturbed Data)									
Model	Accuracy	Recall	F1 Weighted	Cohen’s Kappa	Positive Precision	Negative Precision	Error Rate	Loss	Computing Time (s)
XGBoost	0.75 ± 0.06	0.27 ± 0.09	0.8 ± 0.04	0.04 ± 0.04	0.1 ± 0.03	0.93 ± 0.01	0.25 ± 0.06	0.69 ± 0.0	0.2 ± 0.02
Random Forest	0.92 ± 0.01	0.0 ± 0.0	0.89 ± 0.02	0.0 ± 0.0	0.0 ± 0.0	0.92 ± 0.01	0.08 ± 0.01	0.39 ± 0.01	6.95 ± 0.29
Logistic Regression	0.63 ± 0.02	0.44 ± 0.07	0.72 ± 0.01	0.03 ± 0.02	0.09 ± 0.02	0.93 ± 0.01	0.37 ± 0.02	0.66 ± 0.02	1.08 ± 0.2
Naive Bayes	0.51 ± 0.05	0.62 ± 0.09	0.62 ± 0.05	0.03 ± 0.03	0.09 ± 0.02	0.94 ± 0.02	0.49 ± 0.05	0.79 ± 0.04	0.08 ± 0.0
CNN	0.9 ± 0.02	0.08 ± 0.06	0.88 ± 0.02	0.07 ± 0.07	0.3 ± 0.27	0.93 ± 0.01	0.1 ± 0.02	0.31 ± 0.05	810.6 ± 16.78

Note: CNN shows better performance in Positive Precision (0.3 ± 0.27) and Error Rate (0.1 ± 0.02) for GAD perturbed data.

**Table 7:** Comparison of other studies using ML classifiers for mental health prediction.

Study	Goal	Methods	Input Data	Model Performance	Comparison
The current study	Assess ML models’ reliability for mental health prediction with subjective data.	CNN, XGBoost, Random Forest, Logistic Regression, Naïve Bayes	Self-reported surveys from students (sociodemographics, health, lifestyle)	CNN best. High accuracy, resilience with biased data, specific features’ impact	NA
Single classifier vs. ensemble ML approaches for mental health prediction [24]	Evaluate ML algorithms for mental health prediction.	Logistic Regression, Gradient Boosting, Neural Networks, KNN, SVM, DNN, XGBoost, Ensemble approach	Open data set (OSMI Mental Health in Tech Survey) on mental health in tech industry	Gradient Boosting best, NN also good. Feature selection important (family history, age).	Similar use of ML in a different context (mental health in tech focusing on burnout and anxiety) resulted in different best models like Gradient Boosting for clean data and ensemble approaches for noisy data.
Prediction of Mental Health Problem Using Annual Student Health Survey: Machine Learning Approach [25]	Predict student mental health using health survey responses and response times.	Logistic Regression, Elastic Net, Random Forest, XGBoost, LightGBM	Responses to health surveys (demographics, survey answers, response time)	Elastic Net and LightGBM best, specific survey questions and response times impactful.	Similar use of ML in a different data (health surveys) resulted in different best models like Elastic Net and LightGBM
Predicting Mental Health Problems in Adolescence Using Machine Learning Techniques [22]	Develop a model for predicting mental health problems in adolescence using ML.	Random Forest, XGBoost, Logistic Regression, Neural Network, SVM	Parental report and register data (474 predictors), SDQ for mental health	Random forest and SVM best, but similar performance to Logistic Regression. Parental reports and environment important.	Their study and the current study both identified Random Forest as the best performing model, for data without added error.

## Data Availability

The original data are available on Dryad: https://doi.org/10.5061/dryad.54qt7 (accessed on 9 November 2018) [55]. The processed data used in this study are available on GitHub: http://github.com/wailimku/MDD_GAD.git (accessed on 3 March 2024).
